# Research of wet string grid dust removal vehicle and creation of dust control area on tunnel working face

**DOI:** 10.1038/s41598-024-57748-x

**Published:** 2024-04-09

**Authors:** Huan Deng, Shiqiang Chen, Junxin Huang, Zhirong Wu, Ying Rao, Xinyi Qiu, Jiujun Cheng

**Affiliations:** 1 School of Civil Engineering, Hunan University of Science and Technology, Xiangtan, 411201 Hunan China; 2 School of Chemical and Environmental Engineering, Hunan Institute of Technology, Hengyang, 421000 China; 3 School of Energy and Built Environment, Guilin Institute of Aerospace Technology, Guilin, 541000 Guangxi China

**Keywords:** Drill and blast method tunnel, Wet grid dust removal crawler vehicle, Air cooler outlet, Dust control area, Dust removal, Civil engineering, Environmental sciences

## Abstract

The spread of blast dust throughout the tunnel becomes a common problem in drill and blast tunneling,the key to breaking through the problem is the creation of a dust control area on the working face.In view of this key problem, a wet string grid dust removal crawler vehicle was developed, the power of the vehicle came from the diesel generator, and further, the air cooler of the diesel generator was used to generate airflow, and the suction process formed by the on-board axial flow fan was coupled to create a dust control area of the working face after blasting.The results show that when the frequency of the axial flow fan is adjusted to 30 Hz, the airflow speed of the wet chord grid section reaches 3.34 m/s, and the dust removal efficiency is the highest, with a value of 94.3%.Compared with the non-use of the dust removal vehicle, when the air outlet of the air cooler is front, horizontal front, horizontal rear, the dust concentration is reduced by 74.37, 92.39 and 50.53%.Finally, the optimized wet grid dust removal crawler was installed in the Dading tunnel, and the actual dust reduction efficiency was about 78.49%. The results obtained provide an important technical way to improve the working environment of the drilling and blasting construction tunnel.

## Introduction

For tunnel construction, the drilling and blasting method generates a lot of dust, especially respiratory dust with high mass concentration, and its suppression and treatment technology has been a research difficulty plaguing the industry. On the one hand, dust induces wear and tear on construction equipment, reducing the accuracy of instruments and shortening their lifespan. On the other hand, construction workers are susceptible to pneumoconiosis due to prolonged dust inhalation^1^. According to relevant statistics, the incidence rate of pneumoconiosis among occupational diseases is above 80 percent^2^. Therefore, it is important to provide a favorable working environment for construction workers to alleviate their risk of illness and improve construction efficiency.

Mechanical ventilation is widely used in construction sites to control dust at the working face, and scholars all over the world have adjusted many key parameters in the ventilation system to meet the requirement^3–7^. However, as tunnel excavation tends to grow in size, it is difficult to realize ideal effect of ventilation and dust removal. In the engineering field, dry filtration, foam adsorption, electrostatic capture, negative ion catalytic and many other dust removal technologies have been created to reduce the concentration of dust at the working face and improve the operating environment of the tunnel^8–13^. According to relevant studies, the most economical and effective means of controlling dust is spraying dust. Researchers have focused on active magnetized water spray, pneumatic atomization, and ultrasonic atomization to deal with the issue^15–17^. However, the tunnel dust source is susceptible to airflow turbulence, the fog droplets and dust particles are difficult to contact, the effective distance of wetting and condensation is limited, greatly reducing the efficiency of spray dust reduction. Wetting fine wire air cleaning technology with high dust removal efficiency, low resistance and stable performance and other characteristics, which captures dust effectively by forming a dynamic water film through the wetting and coagulation of spray droplet groups and dust particles, as well as droplet groups on the fine wire surface causes collision, developing into a kind of promising engineering application^18^.

This paper develops a wet chord grid dust removal crawler vehicle for tunnel construction which is on the basis of wetting fine wire air cleaning technology and drill and blast method. At the same time, in order to ensure that the dust at the working face is effectively pumped and purified, a dust control zone in the working face was created by the use of air cooler, and the spread and aggregation of unpurified dust at the secondary lining is effectively prevented^19,20^, so as to maintain the tunnel construction environment. In addition, dust control, dust removal, self-drive and control devices are integrated into the vehicle without negatively affecting the tunnel production operations, which provide a technological approach to improving the operating environment in drill and blast tunneling operations.

## Engineering background

### Engineering overview

**Figure 1:**
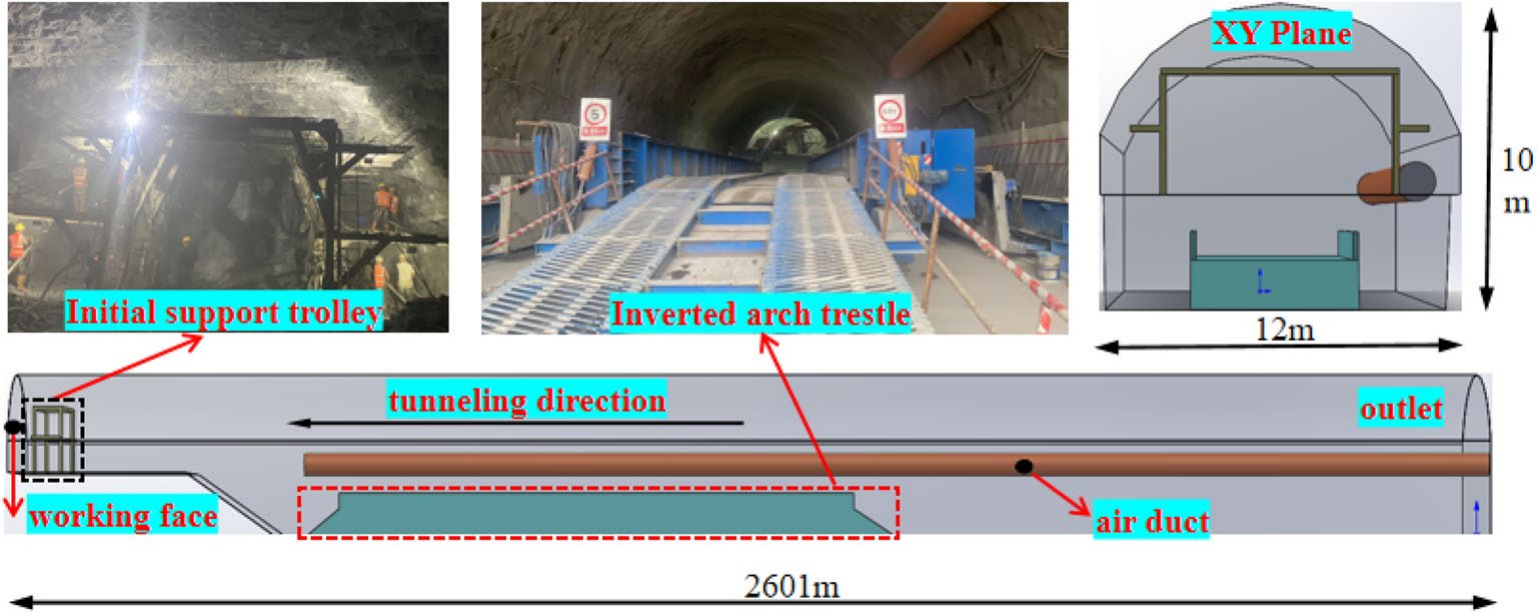
Schematic diagram of on-site construction.

The Dading Tunnel is a single-hole double-lane tunnel with a design length of 2609 m, a width of 12 m, and an arch height of 10 m.According to the grade of the surrounding rock of the tunnel body and the Code for Construction Design of Railway Tunnels^21^, the short step method is selected for excavation, and the step length is 12 m.The tunnel adopts press-in ventilation, the diameter of the air duct is 1.5 m, and the distance from the outlet end to the working face is 20 m, so that the fresh air flow outside the tunnel can reach the working face smoothly.The schematic diagram of on-site construction is shown in Fig. 1.

The construction of the tunnel body mainly includes drilling, blasting, slag removal, support and lining and other processes, and the maximum value of the required air volume should be comprehensively determined by considering the exhaust smoke of the working face, the maximum number of workers in the tunnel, the minimum allowable airflow speed, and the dilution and discharge of exhaust gas of the internal combustion engine. Table 1 shows the required air volume for the tunnel^22^.

**Table 1:** Calculation of air demand of working face under different working conditions.

	Exhaust the smoke of the gun	Maximum number of workers	The minimum permissible airflow speed	Dilution of exhaust gases
Calculations	$Q_{{\text{y}}} = \frac{{7.8}}{t}\sqrt[3]{{G\left( {AL_{{\text{p}}} } \right)_{{}} ^{2} }}$	$Q_{{\text{p}}} = k_{{\text{f}}} mq_{{\text{f}}}$	$Q_{{\text{w}}} = 60{\text{v}}A$	$Q_{{\text{f}}} = q\sum {\text{w}}$
Air demand/(m^3^/min)	1769.4	202.4	769.5	1824.6

As can be seen from Table 1, the maximum air demand of the tunnel is 1824.6m^3^/min, and the air supply volume of the press-in ventilator outside the tunnel is calculated considering the air leakage volume of the press-in ventilation pipe. This air volume is calculated by the following formula:

1 $$Q_{{_{{\text{j}}} }} = PQ_{{_{Z} }}$$

Among them, *Q*_z_ is the air demand of the working face, m^3^/s; *Q*_j_ is the air supply volume of the ventilator, m^3^/s; *P* is the air leakage rate of the press-in duct, %, is calculated as follows:

2 $$P = \frac{1}{{1 - \frac{{L_{{\text{p}}} }}{100}P_{100} }}$$

Among them, *L*_p_ is the length of the pipe, take the longest pipe length, m; *P*_100_ is the average air leakage rate of 100 m, take 1%.

Accordingly, the 2 × 75 kW axial flow fan SDF(C)-NO11.5 is selected to supply fresh air to the tunnel, with a rated air volume of 1171–2285 m^3^/min and an air pressure of 727–4629 Pa, which can meet the ventilation requirements of the unilateral tunnel.

### On-site measurement

**Figure 2:**
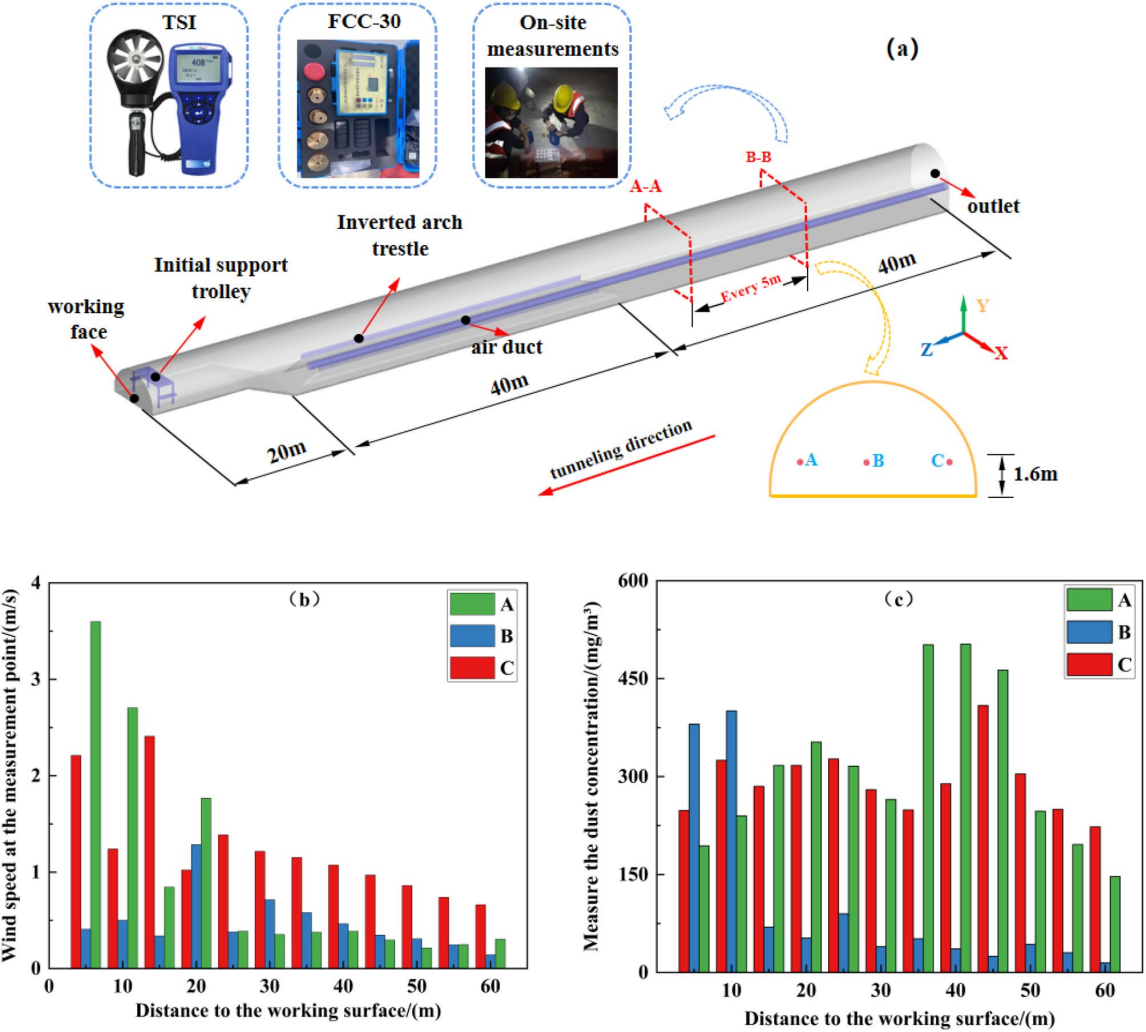
Field airflow speed and dust concentration measurements.

In order to visually analyze the airflow and dust migration in the tunnel, the TSI air speed measuring instrument and the FCC-30 dust measuring instrument are used to measure the three measurement points shown in Fig. 2a, and one group is recorded every 5 m. In the process of measurement, the tunnel is undergoing blasting and smoke exhaust operations, and the airflow speed and dust distribution at the tunnel measuring point are shown in Fig. 2b and c, wherein Fig. 2b is the airflow speed distribution at the tunnel measuring point, and Fig. 2c is the dust distribution at the tunnel measuring point.

It can be seen from Fig. 2b and c that after the air flow is injected out of the press-in air duct, due to the turbulent diffusion and entrapment of the jet, the gun smoke of the working face is strongly mixed with the fresh air, and it is pushed outward along the tunnel.Within 20 m from the working face, the airflow speed of measurement point B is smaller than that of A and C, and there is a vortex in the middle of the tunnel, so the dust concentration of measurement point B is high. In the range of 20–60 m from the working face, the reflux crosses the upper step, mainly moving along one side of the C measuring point, the airflow speed of the C measuring point is larger than A and B, the airflow speed of the measuring point A is the smallest, the dust is retained on the A side, and the dust concentration of each measuring point exceeds the standard value. Therefore, it is urgent to take the necessary dust removal measures.

## Development of wet chord grid dust removal crawler vehicle

### Structure

**Figure 3:**
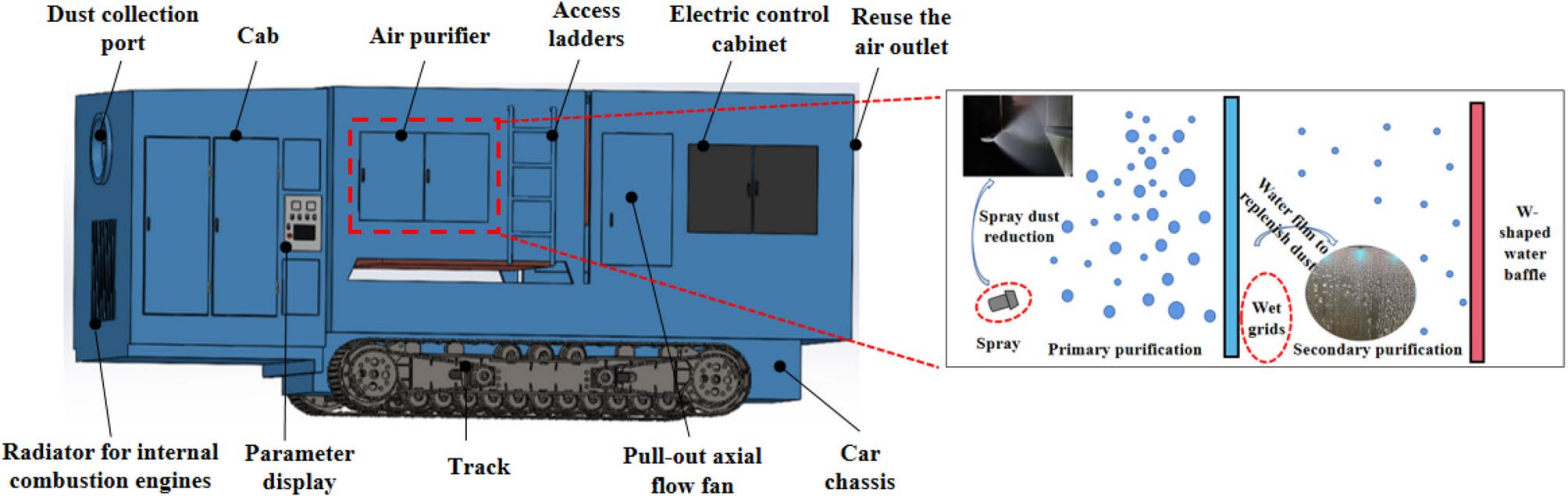
Schematic diagram of the device structure.

In order to improve the tunnel construction environment and reduce the dust concentration in the tunnel, a wet chord grid dust removal crawler vehicle as shown in Fig. 3 was developed, and the dust was reduced at the working face.The wet grid dust removal crawler truck is mainly composed of a drive system (including diesel generators, hydraulic pumps, air cooler, etc.), air purification boxes, and extractable axial flow fans. After blasting in the tunnel, the vertical water pump and the extraction axial flow fan are started through the electric control box, and the dust and dirty air at the working face are purified through the air inlet under the suction action of the extraction axial flow fan of the device, and the reused air is directly discharged into the tunnel to dilute the pollutant concentration in the tunnel and promote the air flow to move outside the tunnel.

### Wet string grid dust removal mechanism

**Figure 4:**
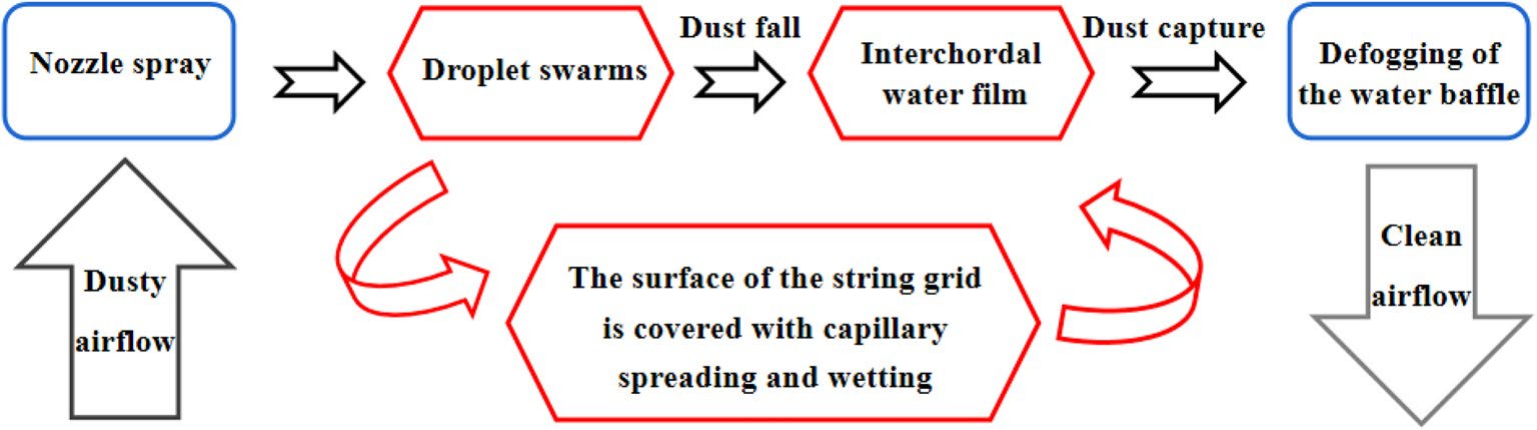
Principle of wet string grid dust removal.

Wet string grid water film dust removal is based on the water film washing and dust removal method based on the combination of dust and water, the string grid is the medium of film making, the water film is equivalent to a barrier, when the dusty air flow through the water film, the washing airflow, dust particles are trapped in the water film, to achieve the purpose of dust removal and purification. In the air purification box, spray, string grid film-making, and water retaining and defogging work together to form a string grid water film dust removal and purification system, as shown in Fig. 4.

Specifically, the droplet group ejected from the nozzle collides with the surface of the string grid by inertia, and the droplets are capillary wetted on the surface of the string grid and the capillary spread between the strings due to capillary action, and the longitudinal fine chord grid gap forms a dynamic water film flowing downward. When dusty air passes through the water film, the dust particles are captured by the water film and flow downward with the water flow. The water film ruptures under the action of airflow, and continuously generates new water film under the action of continuous spray droplets. The water mist formed by the rupture of the string grid is defogged by the water baffle, which further separates the dust contained in the water mist and discharges clean air^23–25^.

### Optimization of the operating parameters of the wet chord grid dust removal crawler vehicle

**Figure 5:**
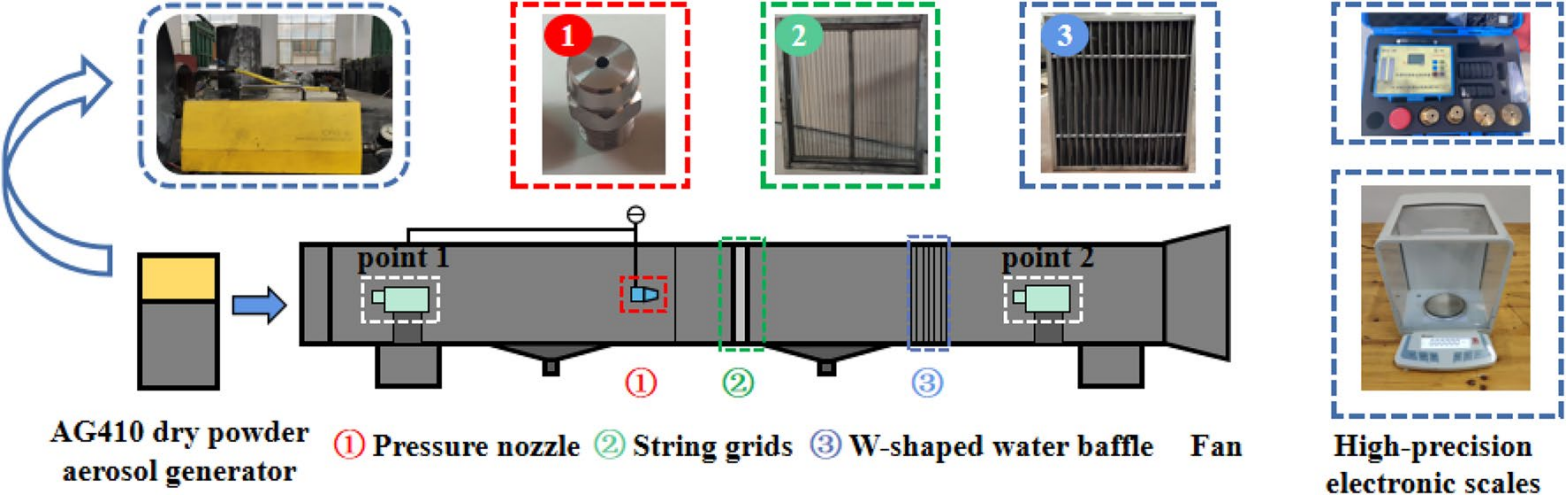
Experimental platform for wet string grid filtration and dust removal.

The formation and crushing of the water film on the surface and between the strings of the wet string grid are mainly affected by the water supply pressure of the nozzle and the filter airflow speed of the string grid section.Due to the limited water supply pressure of the vehicle-mounted water pump, there are many impurities in the water source in the tunnel, and the nozzle with a small aperture is easy to be blocked, and the atomization effect is poor; The nozzle with a large pore size is selected, and the particle size of the spray droplet is too large, and it is difficult to form a water film between the string grids; Finally, a low-pressure atomizing nozzle with an aperture of 2.0 mm was selected, and the dust removal effect was significant when the water supply pressure was 0.6 MPa^18^.Under different fan frequencies, the air volume inhaled by the dust collection port is different, which will lead to the difference in the filtration air speed of the wet chord grid section, which will affect the dust removal efficiency. The airflow speed of the chord grid section under the conditions of 20, 30, 40 and 50 Hz was measured by the TSI air speed measuring instrument, which was 2.85, 3.34, 3.83 and 4.34 m/s, respectively. Figure 5 shows the self-built wet string grid filtration and dust removal experimental platform, which includes AG410 dry powder aerosol diffuser, low-pressure atomization nozzle, string grid, W-shaped water baffle, extraction axial flow fan and dust sampler.

**Figure 6:**
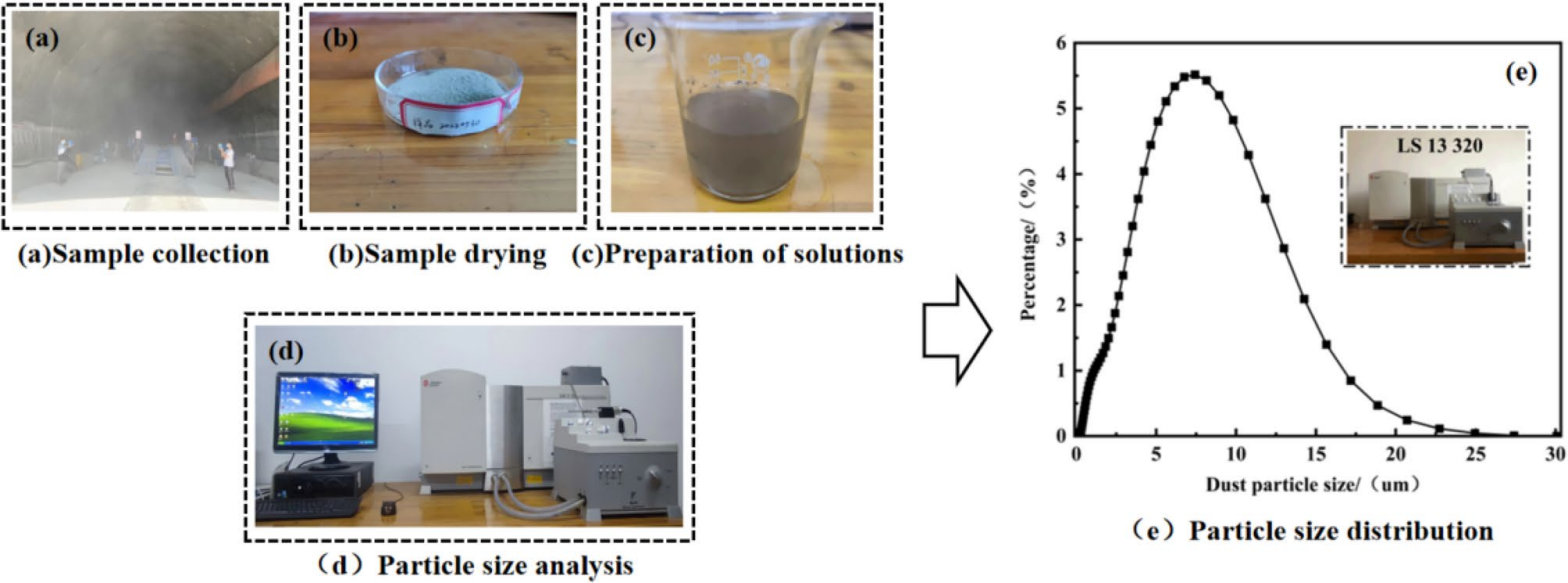
Dust particle size analysis.

As shown in Fig. 6, the dust collected in Fig. 2 is analyzed by LS 13 320 particle size analyzer, and the dust particles are mainly distributed between 2 and 25 μm, with 77.85% of the dust below 10 μm and an average particle size of 8 μm.Therefore, the dust with a particle size of d < 25 μm is screened for experiment, and the AG410 dry powder aerosol diffuser emites dust particles into the pipeline, and two dust sampling points are arranged at the front and rear ends of the wet string grid, after the normal operation of the experimental platform, the sampler can accurately sample the dust before and after the dust removal of the string grid, and the dust removal efficiency is calculated by weighing the filter paper after the dust sampler sampled and dried.

Table 2 shows the dust quality and dust removal efficiency of the measured points at different fan frequencies.

**Table 2:** Dust quality and dust removal efficiency at different fan frequencies.

Fan frequencies/(Hz)	Average airflow speed of the chord grid section/(m/s)	Point1/(g)	Point2/(g)	Dust removal efficiency
20	2.850	2.000	0.236	88.2%
30	3.340	2.000	0.114	94.3%
40	3.830	2.000	0.194	90.3%
50	4.340	2.000	0.250	87.5%

As can be seen from Table 2, when the fan frequencies are 20, 30, 40 and 50 Hz, the dust removal efficiency of the wet string grid is 88.2, 94.3, 90.3 and 87.5%, respectively. When the fan frequency is 30 Hz, the dust removal efficiency can reach up to 94.3%, and when the fan frequency is increased to 40 and 50 Hz, the airflow speed of the chord grid section increases, the water film between the chords is not easy to form, and the dust removal efficiency decreases. The experimental results provide a theoretical basis for the optimization of the operating parameters of the device.

## Creation of dust control area on the working face

Due to the large cross-sectional area of the tunnel, only dust removal measures are taken on the working face, and a large amount of dust is still diffused. Therefore, it is also necessary to create a dust control zone on the working surface. The air outlet of the air cooler of the diesel generator of the dust removal truck is a square of 1 m × 1 m, and the airflow speed is constant at 8 m/s.

### Dust control area construction mechanism

1. Vortex blowing and suction agglomeration dust control technology

**Figure 7:**
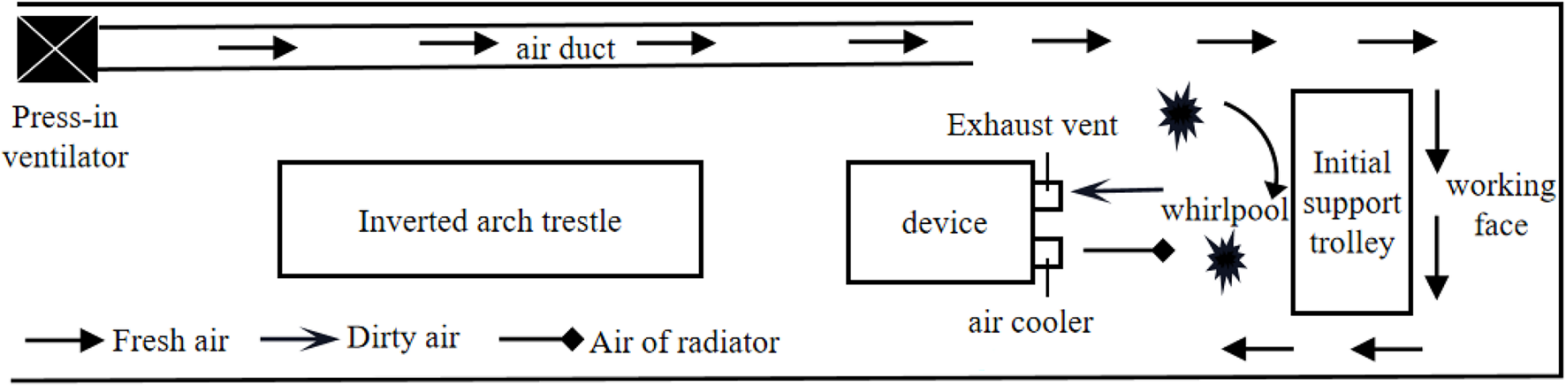
Vortex blowing and suction accumulation dust control.

Figure 7 shows the dust control principle of the pre-installed device of the air cooler. In Fig. 7, the fresh air pressed into the press-in air duct, the heat dissipation outlet air of the device, and the suction air flow of the extractable axial flow fan follow the vortex blowing principle, forming a strong vortex air flow between the working face and the device, and the air flow is finally attenuated and sucked in by the extraction air duct, so as to form a vortex blowing and suction accumulation dust control technology^26^.

(2) Jet air curtain dust control technology

**Figure 8:**
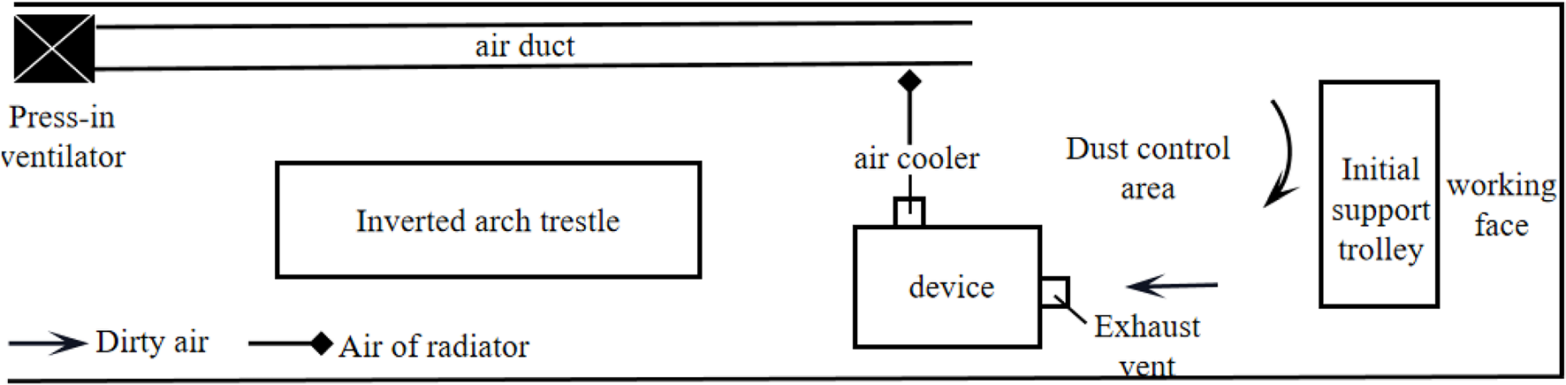
Jet air curtain dust control.

Figure 8 shows the dust control mechanism of the device when the air cooler is horizontally positioned. In Fig. 8, the wet string grid dust removal crawler is installed close to the side wall of the tunnel, the device is opened, the press-in ventilator is closed, and the air outlet of the air cooler is directed to form an air curtain layer at a certain flow rate, and the dust control area is formed by sucking up the surrounding air^27^, and finally, the dirty air in the dust control area is sucked in by the extraction air duct.

### Establish the model and set the parameters

#### Physical Models

**Figure 9:**
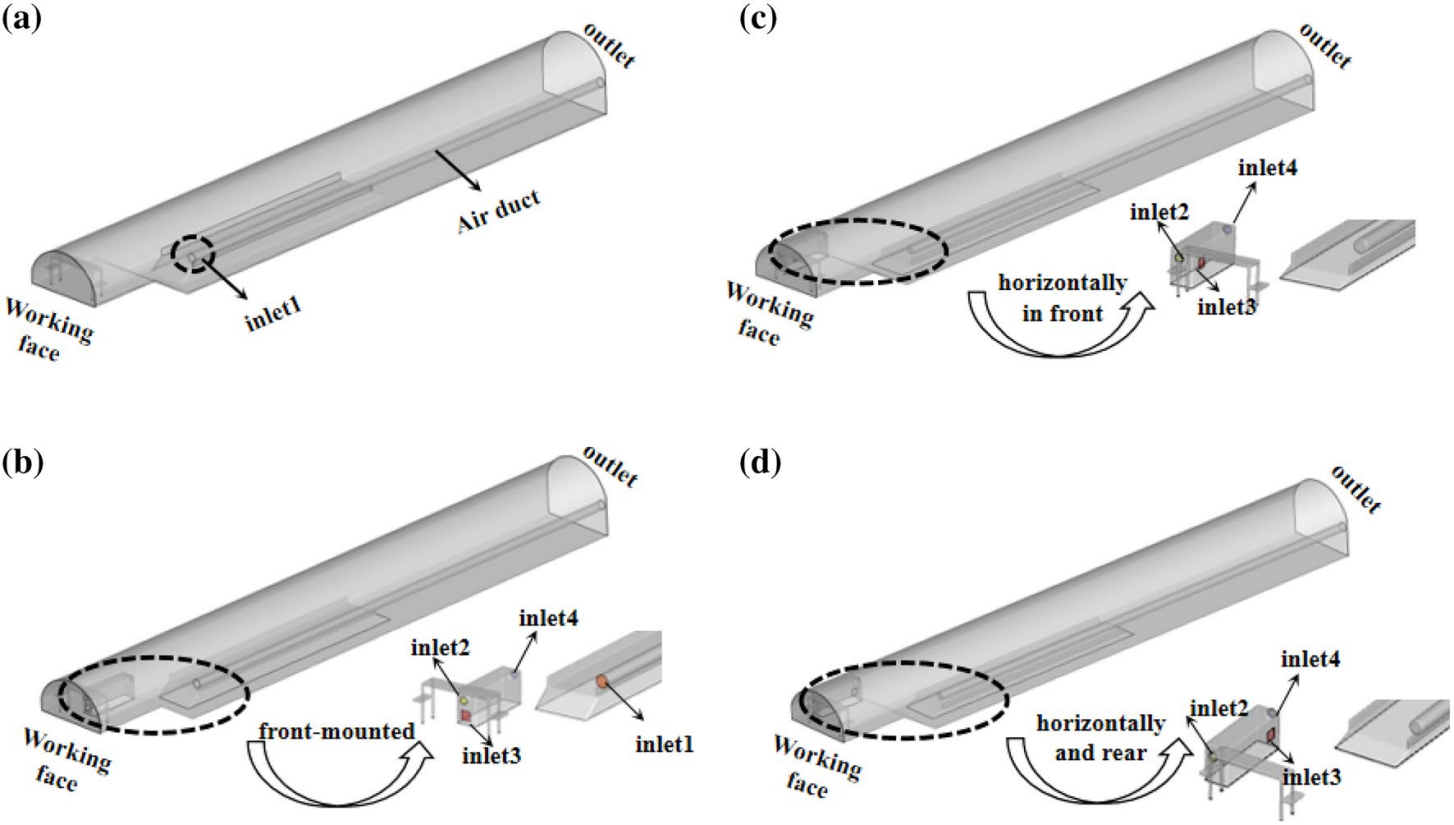
Physical model.

According to the actual working conditions, the physical model as shown in Fig. 9 is established at a scale of 1:1.The model includes a tunnel, a primary support trolley, a wet grating dust removal crawler (8 m long, 2 m wide and 2.2 m high), an inverted arch trestle and a press-in air duct. Wherein, model (a) is a tunnel single-head compressed air model; Model (b) Front-facing air cooler for installation; The devices (c) and (d) of the model are located on the opposite side of the press-in air duct, and the air cooler is placed horizontally in front and rear respectively.

In Fig. 9, the outlet end of the press-in air duct is set to inlet1, the dust collection port is set to inlet2, the air cooler outlet is set to inlet3, and the reuse air outlet is set to inlet4. In order to simplify the calculation, in the simulation process, it is assumed that the air flow after the purification of the wet string grid dust removal crawler vehicle is a clean and dust-free air flow.

#### Mathematical models

The airflow speed in the tunnel is not large and the pressure change is small, so the compressibility of the air can be ignored. Therefore, the air flow in the tunnel is considered as a three-dimensional incompressible and stable viscous turbulence. The model of turbulent flow is a high Reynolds number *k-ε* model. Mathematical models include continuity equations, momentum equations, and *k-ε* model equations^28,29^.

Incompressible Continuity Equation:

3 $$\frac{{\partial \rho \overline{u}_{i} }}{{\partial x_{i} }} = 0$$

Incompressible Momentum Equation:

4 $$\frac{{\partial (\rho \overline{u}_{i} )}}{\partial t} + \frac{{\partial (\rho \overline{u}_{i} \overline{u}_{j} )}}{{\partial x_{i} }} = \rho f_{i} - \frac{\partial p}{{\partial x_{i} }} + \frac{\partial }{{x_{i} }}(\mu \frac{{\partial \overline{u}_{i} }}{{\partial x_{i} }}) - \frac{{\partial (\rho \overline{u^{\prime}}_{i} \overline{u^{\prime}}_{j} )}}{{\partial x_{j} }}$$

Realizable *k-ε* turbulence model:

5 $$u_{t} = \rho C_{\mu } \frac{{k^{2} }}{\varepsilon }$$

*k* equation:

6 $$\frac{\partial k}{{\partial t}} + \overline{u}_{j} \frac{\partial k}{{\partial j}} = v_{t} \left( {\frac{{\partial \overline{u}_{i} }}{{\partial x_{j} }} + \frac{{\partial u_{j} }}{{\partial x_{i} }}} \right)\frac{{\partial \overline{u}_{i} }}{{\partial x_{j} }} - \frac{\partial }{{\partial x_{j} }}\left[ {\left( {\frac{{v_{t} }}{{\sigma_{k} }} - v} \right)\frac{\partial k}{{\partial x_{j} }}} \right] - \varepsilon$$

*ε* equation:

7 $$\frac{\partial \varepsilon }{{\partial t}} + \overline{u}_{j} \frac{\partial \varepsilon }{{x_{j} }} = - C_{{\varepsilon_{1} }} \left( {\partial \overline{u}_{i} \overline{u}_{j} } \right)\frac{{\partial \overline{u}_{i} }}{{\partial x_{j} }} - \frac{\partial }{\partial x}\left[ {\left( {\frac{{v_{t} }}{{\sigma_{\varepsilon } }} - v} \right)\frac{\partial \varepsilon }{{\partial x_{j} }}} \right] - C_{{\varepsilon_{2} }} \frac{{\varepsilon^{2} }}{K}$$

In Eqs. (3)–(7), *ρ* is the fluid density, kg/m^3^; *U*_I_ and *U*_J_ are the velocity components of the fluid, m/s, respectively; *p* is the pressure on the fluid microelement, Pa; *μ* is the dynamic viscosity, Pa·s; *μ*_t_ is the turbulent viscosity Pa·s; *k* is the turbulent flow energy, m^2^/s^2^; *ε* is the dissipation rate, m^3^/s; *σ*_k_ and *σ*_ε_ are the Prandtl numbers corresponding to *k* and *ε* equations, respectively. According to the relevant experiments, the model constants were *C*_μ_ = 0.09, *C*_ε1_ = 1.44, *C*_ε2_ = 1.92, *σ*_k_ = 1.0, and *σ*_ε_ = 1.3.

#### Parameter Settings

The frequency modulation range of the axial flow fan of the wet string grid dust removal crawler vehicle is 0 ~ 50 Hz, and the frequency of the ventilation fan is adjusted to 30 Hz when the numerical simulation is calculated. In order to ensure the accuracy of the simulated values, the TSI anemometer was used to measure the four velocity inlets inlet1, inlet2, inlet3 and inlet4 in Fig. 7, and the measurement results are shown in Table 3.

**Table 3:** Boundary condition parameter settings.

Speed entrance	inlet1/(m/s)	inlet2/(m/s)	inlet3/(m/s)	inlet4/(m/s)
Measurement results	12	− 15	8	25

The particle size range of the dust source is taken according to Fig. 6, and the particle size range is 2–16 μm, and the particle size range is 2–16 μm, and the mass flow rate and other references^30^ are taken for the values, and the parameters of the injection source are set as shown in Table 4.

**Table 4:** Parameters for injection sources.

Ejection source	Material	Particle size distribution	Minimum/large particle size/m	Median particle size/m	Mass flow rate/kg/s	Distribution index	Motion model
Workingface	SiO_2_	R-R	2 × 10–6/1.6 × 10–5	7.57 × 10–6	0.006	3.05	Random orbit

### Airflow and dust migration law

#### Airflow movement law

**Figure 10:**
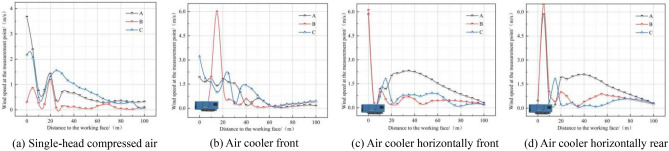
Airflow velocity distribution at human respiration height.

Figure 10 shows the airflow speed distribution at the measuring point at a distance of 1.6 m (human breathing height) from the tunnel floor in the four model tunnels. In Fig. 10a, the tunnel only adopts press-in ventilation, and the air duct directs the air flow along the side wall of measurement point A to the working face, and the jet flows back and spreads along the side wall of measurement point C to the full section. Therefore, in the range of 0–20 m from the working face, the airflow speed of measuring point A is greater than that of measuring points B and C, and in the range of 20–100 m from the working face, the airflow speed of measuring point C is greater than that of measuring points A and B, and the airflow speed of measuring point B in the middle is the smallest.

In Fig. 10b, the device is installed in the middle of the upper step and in front of the air cooler, the air duct and the air cooler respectively draw the air flow to the working face, and the device extraction air duct sucks the air flow into the device, resulting in the disorder of the air flow migration within 0–60 m from the working face. In Fig. 10c, the device is installed on the opposite side of the press-in air duct, and the air cooler is placed horizontally in front, and the press-in ventilator is not turned on at this time, the air cooler is transversely ventilated, and the air flow shoots to the side wall and gradually attenuates, and finally the reused air is discharged along the side wall of the air duct with the purification of the device, and the airflow speed at measurement point A is the largest. In Fig. 10d, the device is installed on the opposite side of the pressed-in air duct, the air cooler is placed horizontally, and other parameters are not changed, and the airflow speed at measurement point A is still the maximum, and compared with Fig. 10c, the airflow speed at measurement point B increases significantly, indicating that changing the installation position of the air cooler will also affect the flow field of the whole tunnel.

#### Dust distribution law

**Figure 11:**
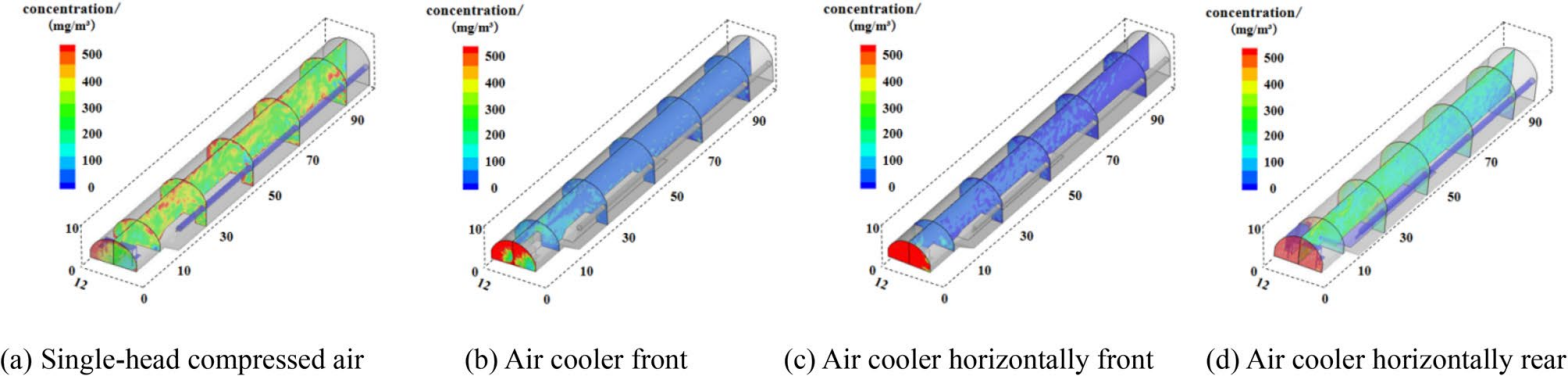
Cloud map of dust concentration distribution in part of the tunnel section.

Figure 11 shows the dust concentration distribution of the tunnel section under the unused device and three air-cooler laying schemes. By observing the dust concentration distribution of X = 6 m (tunnel axial surface), Z = 10 m, Z = 30 m, Z = 50 m, Z = 70 m, Z = 90 m, etc., we can preliminarily judge whether the dust is controlled on the working face, so as to optimize the air-cooler layout scheme.

**Figure 12:**
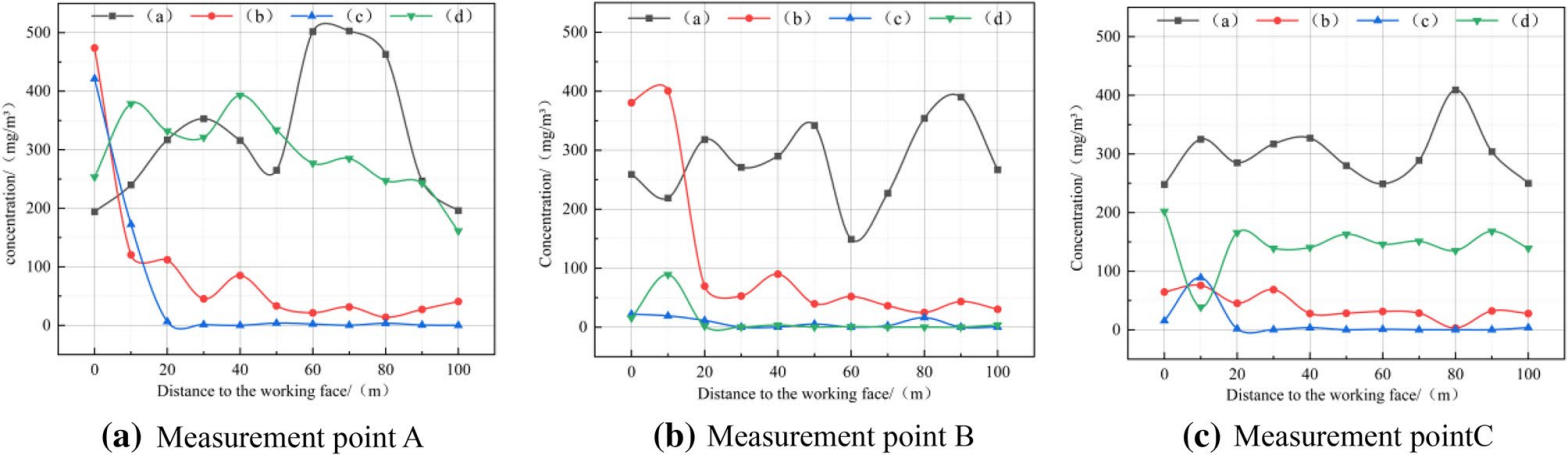
Comparison of dust concentrations at each measurement point under different schemes.

As can be seen from Fig. 11a, the blasting dust was diffused from the working face without the use of the device, polluting the entire tunnel. In Fig. 11b, the air cooler is in front, and the dust is mainly distributed in the area of 0–30 m away from the working face, that is, between the working face and the inverted arch trestle; In Fig. 11c, the air cooler is placed horizontally in front, and the dust is mainly distributed between 0 and 10 m away from the working face, that is, between the dust collection port of the device and the working face; In Fig. 11d, the air cooler is placed horizontally and then released, and the dust fills almost the entire tunnel, which may be due to the fact that the air cooler outlet and the reuse air outlet are too close to each other, and the dust in the dust control area is suctioned by the air cooler outlet and the reuse air flow, which then causes the dust to diffuse to the entire tunnel. As shown in Fig. 12, the dust concentration of each measurement point of human respiration height under different schemes is effectively controlled after the device is adopted.

After the device is adopted, the dust reduction efficiency of different schemes is calculated by Eq. (8):

8 $$\eta = \frac{{C_{{1}} - C_{{0}} }}{{C_{{0}} }} \times {\text{100\% }}$$

where the *η* is the average dust reduction efficiency in the tunnel after the device is adopted, %; *C*_0_ is the average dust concentration in the tunnel when the device is not used, mg/m^3^; *C*_1_ is the average dust concentration in the tunnel after the installation of the device, mg/m^3^.

The results show that compared with the unused device, the dust reduction efficiency is 74.37%. Compared with the unused device, the dust reduction efficiency of the air cooler is 92.39%. Compared with the unused device, the dust reduction efficiency of the air cooler is 50.53%. Therefore, it is better to choose the horizontal front placement scheme of the air cooler, which can create a better dust control area on the working face.

## On-site application

**Figure 13:**
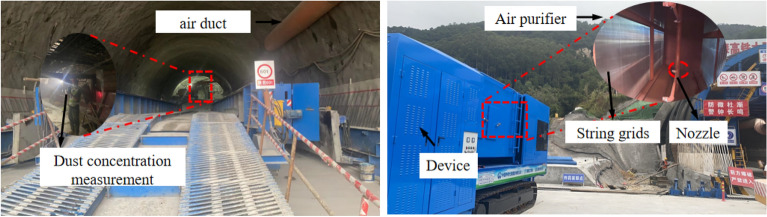
Field application of wet string grid dust removal crawler vehicle.

The wet chord grid dust removal crawler is used in the Dading Tunnel of the Zhuhai-Zhaoqing High-speed Railway in Foshan, Guangdong, as shown in Fig. 13. When the working face is blasted, the press-in fan is not turned on, and the wet string grid dust removal crawler is started. The air cooler transversely directs the air flow to form a dust control area with the working face and the side wall of the tunnel, blocking the full-section diffusion of dust at the upper steps; The extractable axial fan sucks the dirty air in the dust control area into the air purification box, and the purified reused air is directly discharged into the tunnel through the air outlet.

**Figure 14:**
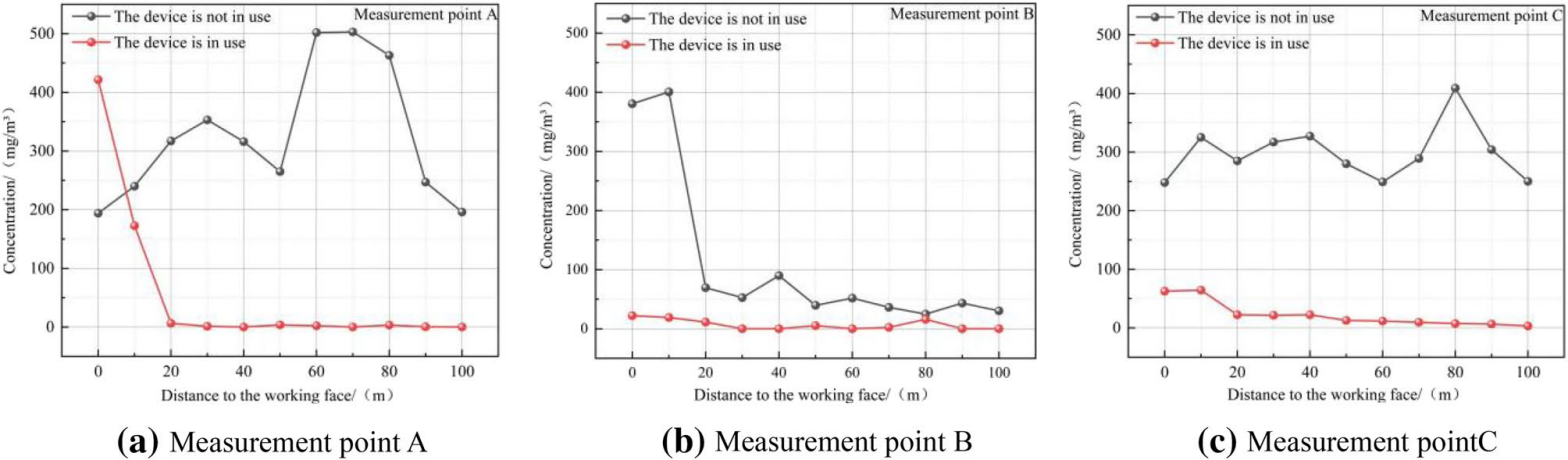
Comparison of dust concentration in the tunnel before and after the installation of the wet string grid dust removal crawler vehicle.

As shown in Fig. 14, after the wet string grid dust removal crawler is installed and operated for a period of time, it is sampled and detected by the FCC-30 dust sampler, and it is shown that a good dust removal effect has been obtained. Before installing the wet string grid dust removal crawler vehicle, the dust concentration in the tunnel is 45–500 mg/m^3^, which is much higher than the national standard requirements; After the installation of the wet string grid dust removal crawler vehicle, the dust mainly gathers around the dust collection port of the device, and the dust concentration is significantly reduced within the range of 20–100 m from the working face. According to Eq. (8), the dust reduction efficiency after the installation of the device is 78.49%, and the dust reduction effect is remarkable, and the air flow after dust removal and purification basically meets the requirements of the air quality of the tunnel inlet air flow.

## Conclusion

In this paper, with the main goal of reducing the dust concentration in the tunnel and optimizing the construction environment, the performance parameters of dust removal efficiency were optimized on the experimental platform, and the layout scheme of the air cooler outlet was compared and selected through numerical simulation, and the dust control area was created at the working face. Based on the obtained results, a wet grid dust removal crawler was designed and developed and applied in the field. Specifically, the following conclusions can be obtained:

1. In order to prevent the diffusion of dust at the tunnel working face and deteriorate the construction environment, a wet string grid dust removal crawler vehicle was developed, and the dust reduction efficiency of field application was 78.49%, which provided an important technical way to improve the tunnel construction environment.
2. When the frequency modulation of the extractable axial fan is 30 Hz, the airflow speed of the wet chord grid section is 3.34 m/s, and the dust removal efficiency is up to 94.3%.
3. The air cooler outlet layout scheme of the device was compared and selected through the numerical model, and the dust reduction efficiency of the air cooler was 74.37, 92.39 and 50.53% respectively compared with the unused device.

## Data Availability

Data will be made available on request.

## List of symbols

*Q*_z_: The air demand of the working face (m^3^/s)
*Q*_j_: The air supply volume of the ventilator (m^3^/s)
*P*: The air leakage rate of the press-in duct (%)
*L*_p_: The length of the pipe, take the longest pipe length (m)
*P*_100_: The average air leakage rate of 100 m (%)
*ρ*: The fluid density (kg/m^3^)
*U*_I_ & *U*_J_: The velocity components of the fluid (m/s)
*p*: The pressure on the fluid microelement (Pa)
*μ*: The dynamic viscosity (Pa s)
*k*: The turbulent flow energy (m^2^/s^2^)
*ε*: The dissipation rate (m^3^/s)
*σ*_k_ & *σ*_ε_: The Prandtl numbers corresponding to *k* and *ε* equations
*η*: The average dust reduction efficiency in the tunnel after the device is adopted (%)
*C*_0_: The average dust concentration in the tunnel when the device is not used (mg/m^3^)
*C*_1_: The average dust concentration in the tunnel after the installation of the device (mg/m^3^)
